# Quality Evaluation Using the Mobile App Rating Scale for Speech Therapy in Parkinson Disease: Systematic Search and Evaluation

**DOI:** 10.2196/72214

**Published:** 2026-08-24

**Authors:** Tooba Haris, Harshvi Shah, Jose F Florez-Arango

**Affiliations:** 1 Department of Population Health Sciences, Weill Cornell Medicine, 1300 York Ave, New York, NY, 10065, United States, 1 646 962 8001; 2 Center of Biomedical Informatics and Biostatistics (CB2), University of Arizona, Tuscon, AZ, United States

**Keywords:** Parkinson disease, mobile apps, mobile health, speech therapy, MARS, Mobile App Rating Scale, PRISMA

## Abstract

**Background:**

As the global population ages, Parkinson disease (PD) has emerged as the second most prevalent neurodegenerative condition after Alzheimer disease. People with PD often experience speech problems, including reduced volume, monotone pitch, breathiness, and word slurring. Interventions such as speech therapy through mobile apps provide reassurance and easier access to care for patients having this neurodegenerative condition. Mobile apps offer patients with PD greater access to care and the reassurance of being able to manage their condition at home. However, we do not know the quality of these apps. A systematic evaluation of these mobile apps is necessary to ensure their effectiveness and suitability for use by patients with PD.

**Objective:**

This study aimed to evaluate the quality of existing free apps that support speech therapy for people with PD.

**Methods:**

This study took place at Weill Cornell Medicine in New York, New York. It was conducted between January 2024 and July 2025. We conducted a systematic application search to identify apps available on the Apple App Store for speech therapy targeting people with PD and then performed an evaluation using the Mobile App Rating Scale framework with 4 raters. We only included freely available apps. We calculated interrater reliability and found median and mean ratings and SD for Mobile App Rating Scale dimensions.

**Results:**

From 33 candidate apps, we included 3 apps in our evaluation. Functionality scored high for all 3 apps, with ratings above 4.5. However, engagement scored the lowest in all the apps. Interrater reliability agreement varied for all sections. *Voicibel* had the highest agreement of 0.83 for engagement. Aesthetics had the lowest agreement in all 3 apps, with agreements at 0.33 or below.

**Conclusions:**

All 3 apps offer targeted exercises and are targeted for the patient. Mobile apps hold promise for increasing the accessibility of speech therapy for patients with PD and provide promising support for managing speech difficulties in PD. Evaluating these apps is essential to determine their quality in supporting these patients. Future research with speech therapy apps should evaluate clinical efficacy through randomized controlled trials.

## Introduction

Parkinson disease (PD) is a growing concern as the global population ages. Early diagnosis is crucial, but over 40% of patients miss out on specialized care during this critical phase [1]. PD is a progressive neurodegenerative disorder characterized by a decline in motor function [2]. The hallmark pathology of PD is the loss of dopamine-producing neurons within the substantia nigra pars compacta, leading to the cardinal motor symptoms of resting tremor, rigidity, and bradykinesia [3]. Although the cause of PD remains idiopathic in most cases, genetic and environmental factors are likely culprits, with mutations in specific genes and exposure to certain toxins increasing the risk of developing the disease [4].

PD can significantly impact speech and communication. People with PD often experience speech problems, including reduced volume, monotone pitch, breathiness, and slurring of words [5]. These changes stem from the same underlying issue affecting movement: the loss of dopamine neurons in the brain [6]. Speech therapy plays a crucial role in managing these symptoms. Therapists use techniques such as the Lee Silverman Voice Therapy program to improve vocal loudness, articulation, and breath control [7]. This evidence-based intervention helps individuals with PD maintain clear and intelligible communication, enhancing their quality of life.

Speech and language therapy can improve patients’ vocal quality and help them develop coping strategies to manage their pitch. This could result in patients becoming more confident in their speech production. Pitch therapy may include assessments, reports, therapy programs, reviews, training, advice, and education. Pitch disorders can most appropriately be described as a characteristic of dysphonia, which refers to any change to the vocal quality, leading to a partial loss of voice [8]. We aimed to include apps that target PD specifically to ensure that these challenges were addressed through therapy.

Virtual therapy through mobile apps offers a promising approach to delivering speech therapy to individuals with PD. These apps provide a cost-effective solution for patients to access rehabilitation. Such apps may increase the intensity of improvement, leading to greater long-term recovery [9]. Mobile apps offer patients with PD greater access to care and the reassurance of being able to manage their condition at home.

These apps can be a valuable asset in supplementing speech therapy for PD. They can provide patients with convenient tools for practicing exercises and maintaining progress during therapy sessions. Some apps offer gamified exercises that target specific speech aspects, such as articulation and vocal loudness, making practice more engaging [10]. Additionally, apps can offer visual prompts and reminders to complete exercises throughout the day, promoting consistency in practicing techniques learned in therapy [11]. This ongoing practice can solidify the therapeutic benefits and enhance the long-term effectiveness of speech therapy in managing speech limitations associated with PD. However, it should be noted that mobile apps lack the personalized touch of a qualified speech-language pathologist. Therapists can tailor exercises to individual needs, offer real-time feedback on technique, and track progress over time. Mobile apps serve as a complementary tool, not a replacement, for the expertise and guidance provided by a speech therapist in managing speech symptoms associated with PD.

A systematic evaluation of these mobile apps is necessary to ensure their quality for use by patients with PD. This study examines existing apps using the Mobile App Rating Scale (MARS) evaluation for speech therapy mobile apps targeting people with PD. The MARS framework provides a standardized approach to evaluating mobile health interventions [12]. We used the MARS framework to assess speech therapy apps for PD to gain valuable insights into the strengths, weaknesses, and overall quality of these technologies.

## Methods

### Study Design

This study took place at Weill Cornell Medicine in New York, New York. This study was initially conducted between January 2024 and July 2025. There were 2 screeners who independently identified the apps.

To follow our framework, we had 4 raters in this study. Two raters were the same individuals who participated in the app search. The reviewers were health care professionals, researchers, and students in a health informatics master’s program. The reviewers were familiar with the MARS framework and worked independently.

To identify apps, we searched for the terms “Parkinson’s,” “Parkinson’s disease,” and “speech therapy.” We entered these terms into the search engine on the Apple App Store. From the results, relevant apps were selected for the screening process. We used only apps available on the Apple App Store due to our access being limited to this platform.

### Inclusion Criteria

We used the PRISMA (Preferred Reporting Items for Systematic Reviews and Meta-Analyses) framework (Checklist 1) to identify applications for evaluation [13]. For the search, we identified apps for speech therapy. Before screening, we filtered this search only to include apps specifically targeted toward speech therapy for PD. Two screeners reviewed these apps together to match our inclusion and exclusion criteria. Our inclusion criteria are that apps (1) are available on the Apple App Store, (2) are free to use and do not require a subscription fee, and (3) specify that they are targeted toward speech therapy for individuals with PD.

### Quality Assessment

The MARS framework offers a structured approach to evaluating mobile health apps [14]. This framework helps assess speech therapy apps targeted at patients with PD. The MARS framework considers various aspects of the app’s design, functionality, and usability. It evaluates factors such as ease of navigation, accessibility features for users with motor impairments, the suitability of therapeutic content for PD-specific speech issues, and the app’s ability to track progress over time. By applying the MARS framework, researchers can comprehensively assess whether speech therapy apps for PD meet the specific needs of this patient population. We had 4 reviewers to evaluate the final 3 apps following MARS guidelines. Our evaluation form included 4 sections: engagement, functionality, aesthetics, and information.

The median MARS scores allow us to assess the quality of the 3 apps. Based on literature, a median score of ≥4.0 (IQR 3.45-4.55) demonstrated high quality, a score of 3.0 to 3.99 (IQR 2.6-4.4) displayed moderate quality, and a score less than 3 (IQR 2.8-3) was indicated as poor quality.

The engagement section aimed to evaluate the apps’ engagement with their target audience. Evaluators were asked a series of 5 questions to evaluate entertainment, interest, customization, interactivity, and the target group. The responses were rated on a 5-point scale, with 1 indicating “not entertaining” and 5 indicating “entertaining.” For this section, the following questions were asked:

- Is the app fun/entertaining to use? Does it use any strategies to increase engagement through entertainment (eg, through gamification)?
- Is the app interesting to use? Does it use any strategies to increase engagement by presenting its content in an interesting way?
- Does it provide/retain all necessary settings/preferences for app features (eg, sound, content, notifications, etc)?
- Does it allow user input, provide feedback, and contain prompts (reminders, sharing options, notifications, etc)? Note: these functions need to be customizable and not overwhelming in order to be perfect.
- Is the app content (visual information, language, and design) appropriate for your target audience?

The functionality section evaluated the app’s functionality and usability. This aimed to observe whether the app was easy to learn and whether the instructions and design were intuitive. This time, it was a series of 4 questions with a 5-point rating scale. The scale varied between each question, following the same logic: 1 (“the most negative response”) and 5 (“the most positive response”). For this section, the following questions were asked:

- How accurately and fast do the app features (functions) and components (buttons/menus) work?
- How easy is it to learn how to use the app; how clear are the menu labels/icons and instructions?
- Is moving between screens logical/accurate/appropriate/uninterrupted; are all necessary screen links present?
- Are interactions (taps/swipes/pinches/scrolls) consistent and intuitive across all components/screens?

The aesthetics section observed the apps’ visual appeal, style, and graphic design. There were 3 questions on a 5-point scale, ranging from 1 (“no appeal or poor design”) to 5 (“attractive and high quality”). For this section, the following questions were asked:

- Are the arrangement and size of buttons/icons/menus/content on the screen appropriate or zoomable if needed?
- How high is the quality/resolution of graphics used for buttons/icons/menus/content?
- How good does the app look?

The information section evaluated the information, including text and feedback, provided by the app and its credibility. There was a series of 7 questions, all except one, on a 6-point scale, ranging from 1 (“not available”) to 6 (“highly relevant and logical”). For this section, the following questions were asked:

- Does the app contain what is described in the app store?
- Does the app have specific, measurable, and achievable goals (specified in the app store description or within the app itself)?
- Is the app content correct, well-written, and relevant to the goal/topic of the app?
- Is the extent of coverage within the scope of the app and comprehensive but concise?
- Is the visual explanation of concepts—through charts/graphs/images/videos, etc—clear, logical, and correct?
- Does the app come from a legitimate source (specified in the app store description or within the app itself)?
- Has the app been trialed/tested; must it be verified by evidence (in published scientific literature)?

The MARS framework has been validated with the intraclass correlation coefficient [14]. After obtaining their responses, we calculated interrater reliability (IRR). IRR is the measure of agreement between the raters [15]. Agreement interpretations were based on the Landis and Koch Fleiss κ framework [16]. IRR was calculated to measure the consistency of the rating between the raters. We started by calculating a median score for each section for each reviewer. Median scores were also later used to assess the quality of the apps within each MARS domain. Means and SDs were calculated to observe data trends. Furthermore, the mean of the 4 median domain scores was calculated for each app to allow for the overall app quality comparison. We then calculated the agreement between the 4 reviewers. A reviewer’s median score was compared to the other reviewers’ median scores for that section. If the median scores between the 2 reviewers differed by less than 0.5, they were assigned an outcome of 1. If the median scores were greater than 0.5, they were assigned an outcome of 0. Once all outcomes were allocated for a section, we calculated the agreement by computing the average of the outcomes. An agreement score of 0 meant that there was no agreement between the reviewers. An agreement score of 1 indicated total agreement between the reviewers. This was performed to observe consensus for each section of the 3 individual apps. In cases of low agreement, we retained the scores and interpreted the results with vigilance.

## Results

### App Identification

**Figure 1.**
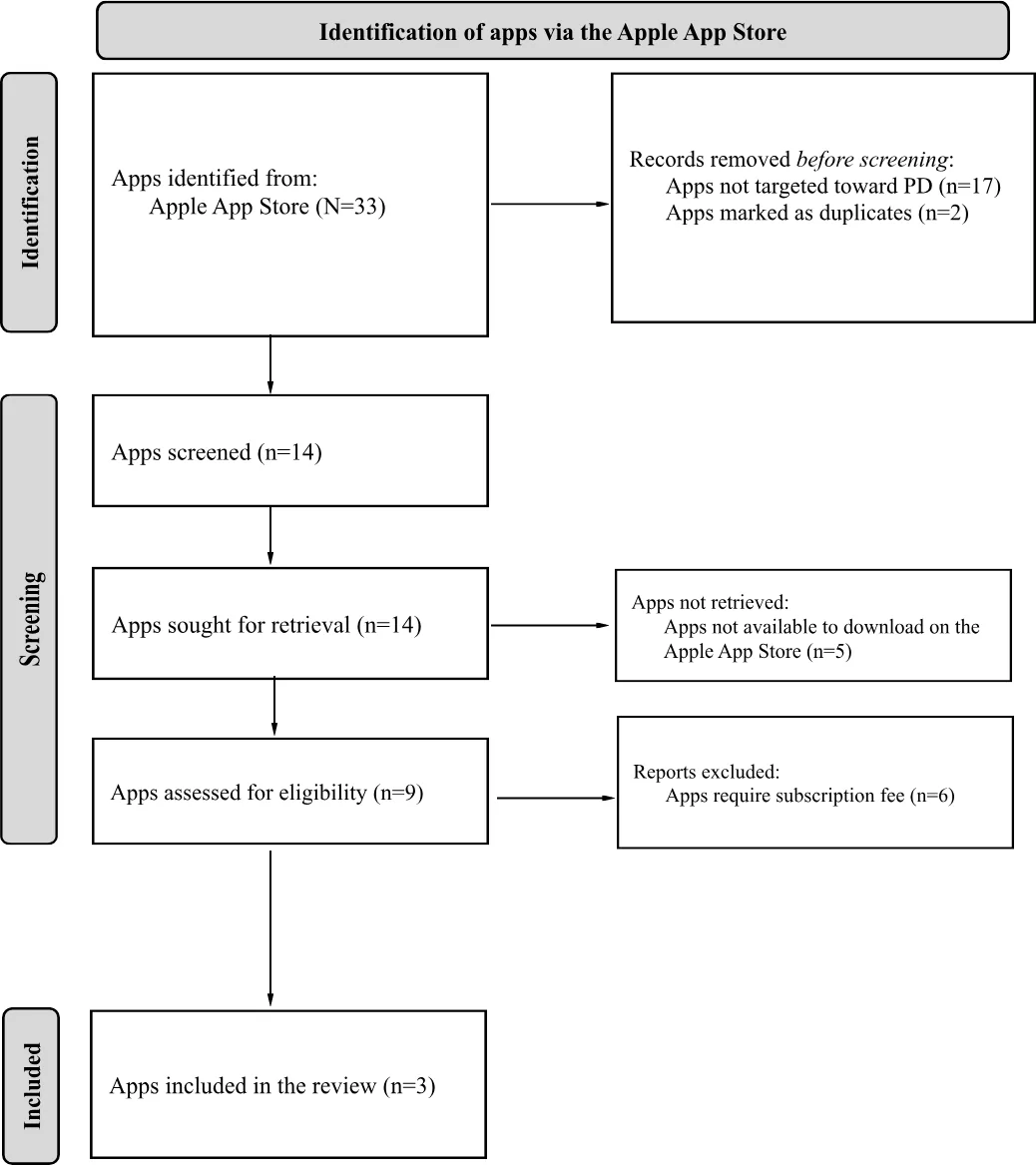
PRISMA (Preferred Reporting Items for Systematic Reviews and Meta-Analyses) framework to find apps included in this review.

On January 20, 2024, we conducted a search on the Apple App Store. Figure 1 uses the PRISMA framework to illustrate the selection process of the apps. We initially identified apps that were searched on the Apple App Store (N=33). We filtered from these to remove apps that were not targeted toward speech therapy for PD (n=17) and duplicates from different searches (n=2). We screened apps and sought them for retrieval (n=14). We identified apps that were unavailable for download on the Apple App Store (n=5) and removed them from our screening process. These apps did not display any buttons for installation. We assessed apps for eligibility (n=9) and excluded those that required a subscription fee (n=6). We finally included 3 apps in our review. Following these criteria, we identified 3 apps for review: *Loud and Clear*, *Speak Up For Parkinson’s*, and *Voicibel*.

### App Description

*Loud and Clear* begins speech therapy with a warm-up before starting the exercises. This includes making phonetic sounds for a few seconds. The app measures the user’s volume using an annotated color-sensitive scale with 3 sections: too soft, in the zone, and too loud. *Loud and Clear* provided videos before beginning the warm-up to demonstrate. Before starting the exercise, it gave instructions on mic distance (12‐18 inches) for accurate measurements. Once again, these instructions were relayed through a video as well. The exercise included reading aloud in a clear and loud voice. The app measured the person’s volume again. Another exercise included answering prompts in complete sentences. It also had an exercise for tongue twisters. The app recommends that the user perform 1 session per day. The session included a warm-up and 3 exercises. It takes less than 10 minutes per day, as the app mentions. It also allowed the user to set reminders, times, and frequency (by enabling them to select days) to notify them when to practice for their next session. It also included a homework section to practice on your own time. *Loud and Clear* provides links to mobile resources for people diagnosed with PD. It included videos on how the app works and its different features. It also contains videos on breathing and speaking guidance, as well as advice on microphone distance, volume control, and pitch adjustment. A particular section of the app enables the user to evaluate their progress. It began with 3 exercises. As they continue using the app, they can use the progress tab again to redo the exercises. In the end, the progress is compared to the baseline recordings of the user and their current recording to allow the user to observe their progress.

*Speak Up For Parkinson’s* allowed people diagnosed with PD to practice their speech. It allowed practice with “word & phrases” or “reading & conversation.” “Words & phrases” provided the user with random words and phrases to say aloud. This app also measured their volume with a bar, which they can observe for improvement. The bar was color-coded red, yellow, and green, with a target zone in the green. At the end of the exercise, the user could watch their video to review their performance. “Reading & conversation” had complete sentences with a similar volume indication bar. Additionally, the app provided speaking tips. It also included a section for donating to the foundation associated with this app.

*Voicibel* aims to check the voice range as a monitoring tool. The interface begins with the patient selecting their desired sound intensity in decibels (dB). They are able to select from 75 dB+, 80 dB+, 85 dB+, 70 to 90 dB, and 80 to 100 dB. For custom settings, the user chooses the measure of voice range to be average or at peak. Lastly, the target type allows the user to create a goal for the exercise. The target includes minimum, range, and offset values. Minimum enables the user to set a minimum decibel that the user must meet. The range allows the user to set a custom target maximum and minimum decibel levels. Lastly, the offset makes the user set a target maximum decibel and an equivalent plus and minus range from the target. While performing the exercise, the visual on the screen is yellow until the targeted intensity is reached. It then turns green. The user can continue practicing the exercise in the same setting for as long as they want. Once they hit the goal and the screen turns green, it resets to yellow, allowing the user to reuse the setting until they stop the session.

On the Apple App Store, *Loud and Clear* had an overall user rating of 3.4 out of 5. This average included 21 ratings. *Speak Up For Parkinson’s* had a rating of 2 out of 5. This low rating may be biased since there are only 3 ratings for this app. *Voicibel* had a rating of 5 out of 5. Based on the reviews, users found *Loud and Clear* to be helpful in improving their voice when used regularly but often encountered issues with glitches in the app. Users found *Speak Up For Parkinson’s* incredibly difficult to use due to issues with accessing the microphone functions in the app. Due to the relatively new nature of *Voicibel*, there are no user reviews available. Table 1 summarizes the characteristics of *Loud and Clear*, *Speak Up For Parkinson’s*, and *Voicibel*. None of the apps were associated with a trial, nor were they tested. They were released through their respective developers.

**Table 1.** Characteristics of the 3 apps.

Characteristics	*Loud and Clear*	*Speak Up For Parkinson’s*	*Voicibel*
App version	6.1.0	1.2.1	0.0.11
Last update	March 2024	March 2023	April 2023
Developer	Darroh LLC	Sandcastle	TD Tech Link LLC
Size	82.2 MB	2.7 MB	33.6 MB
Cost	Free	Free	Free
Rating (out of 5)	3.4 (n=21)	2 (n=3)	5.0 (n=4)
Platform compatibility	iPhone, iPad, iPod, and Mac	iPad and Mac	iPhone, iPad, iPod, Mac, and Apple Vision
Age group	General (4+ years old)	General (4+ years old)	General (4+ years old)

### Quality Assessment

The engagement section for all 3 apps was scored low. *Loud and Clea*r and *Speak Up For Parkinson’s* had moderate quality at 3.0 and 3.8, respectively. *Voicibel* had the lowest score at 2.8. The functionality aspect showed high scores for all 3 apps. For the aesthetics section, *Loud and Clear* and *Speak Up For Parkinson’s* had high scores at 4.2 and 4.0, respectively. *Voicibel* had a moderate score at 3.5. The information section showed a high score for all 3 apps.

The overall mean of the 4 median scores for the apps showed the following: *Loud and Clear,* mean 4.1, SD 0.68; *Speak Up For Parkinson’s*, mean 4.2, SD 0.30; and *Voicibel*, mean 3.8, SD 0.77. The mean scores for each individual section for each app were within 0.4 of the median scores.

**Figure 2.**
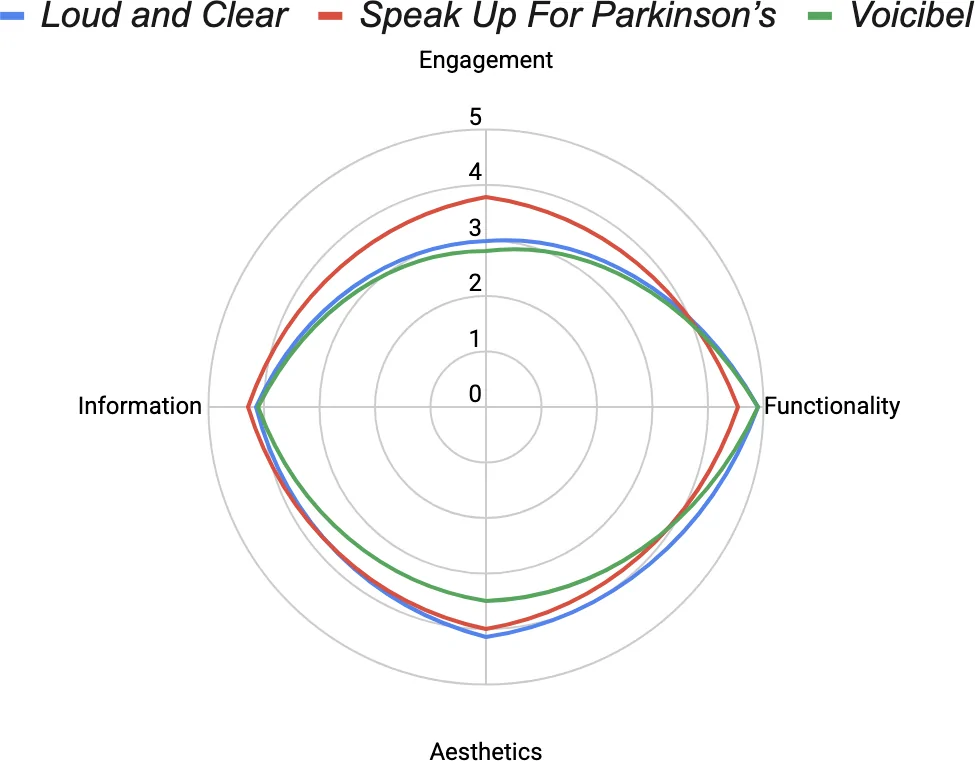
Median ratings of *Loud and Clear*, *Speak Up For Parkinson’s*, and *Voicibel* based on engagement, functionality, information, and aesthetics.

**Figure 3.**
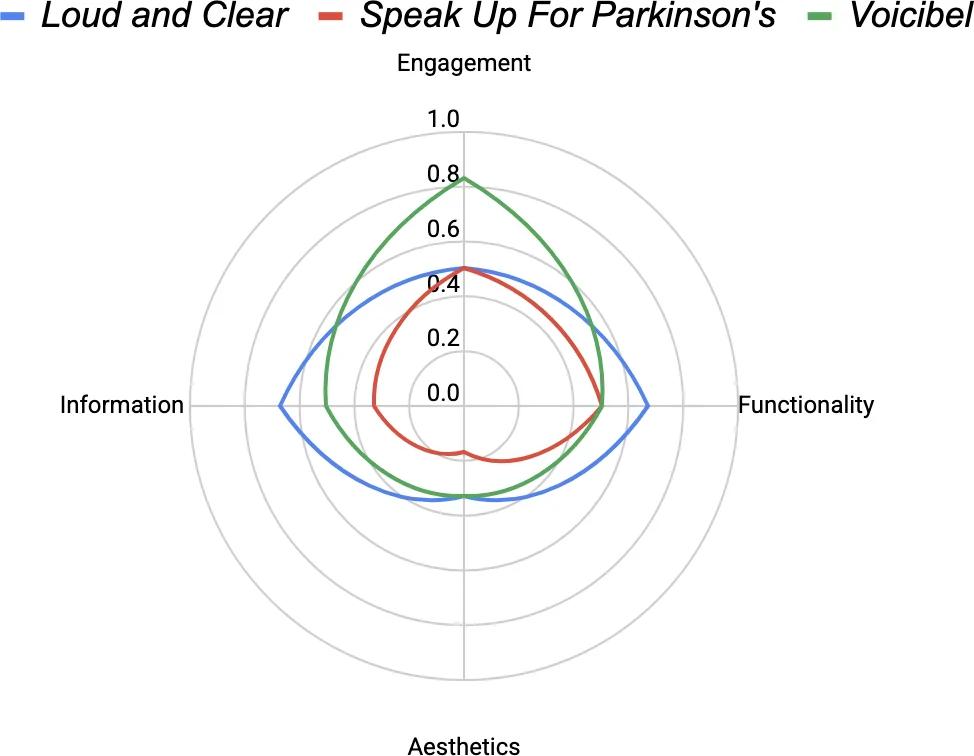
Agreement ratings of *Loud and Clear*, *Speak Up For Parkinson’s*, and *Voicibel *based on engagement, functionality, information, and aesthetics.

We calculated the IRR for each section of all 3 apps based on their ratings (see individual reviewer ratings in Multimedia Appendix 1). Table 2 shows the apps’ median and mean ratings, SD, the calculated agreement, and the average of the agreements. The data are presented in different ways to facilitate interpretation. Figures 2 and 3 show graphical representations of the median ratings and agreement. For most sections in the MARS framework, there was moderate agreement among raters at 0.5. This included the engagement section for *Loud and Clear* and *Speak Up For Parkinson’s*. There was a majority agreement at 0.83 for *Voicibel*. Similarly, for functionality, there was moderate agreement at 0.5 for *Speak Up For Parkinson’s* and *Voicibel,* and majority agreement at 0.67 for *Loud and Clear*.

**Table 2.** Mobile App Rating Scale (MARS) median and mean (*x̄*) ratings, SD, and interrater reliability (IRR) agreement for *Loud and Clear*, *Speak Up For Parkinson’s*, and *Voicibel*.

Criteria	*Loud and Clear*			*Speak Up For Parkinson’s*			*Voicibel*			
	Median (IQR)	*x̄* (SD)	IRR	Median (IQR)	*x̄* (SD)	IRR	Median (IQR)	*x̄* (SD)	IRR	Average IRR
Engagement	3.0 (3.0-3.3)	3.3 (0.60)	0.50	3.8 (3.4-4.0)	3.6 (0.66)	0.50	2.8 (2.8-2.9)	2.9 (0.25)	0.83	0.61
Functionality	4.9 (4.7-5.0)	4.8 (0.35)	0.67	4.6 (4.2-4.9)	4.5 (0.46)	0.50	4.9 (4.4-5.0)	4.5 (0.84)	0.50	0.56
Aesthetics	4.2 (3.9-4.5)	4.3 (0.57)	0.33	4.0 (3.5-4.3)	3.8 (0.78)	0.17	3.5 (3.2-4.0)	3.7 (0.72)	0.33	0.28
Information	4.2 (4.0-4.4)	4.2 (0.27)	0.67	4.3 (3.9-4.7)	4.3 (0.52)	0.33	4.1 (3.8-4.3)	3.9 (0.47)	0.50	0.50

There was a very low level of agreement among raters, at 0.17, for the aesthetics sections of *Speak Up For Parkinson’s*. Whereas *Loud and Clear* and *Voicibel* had fair agreement at 0.33. For the information section, the agreement scores varied among the 3 apps. *Loud and Clear* had a majority agreement at 0.67. *Speak Up For Parkinson’s* had fair agreement at 0.33. Lastly, *Voicibel* had moderate agreement at 0.5. Overall, the aesthetic criteria had very low agreement at 0.28. Functionality and information had moderate agreement at 0.56 and 0.50, respectively. Lastly, engagement had high agreement at 0.61.

On a scale of 1 to 5, the reviewers viewed the subjective quality of all 3 apps as average (3). The subjective quality for *Speak Up For Parkinson’s* and *Loud and Clear* indicated that reviewers would recommend this app to people who might benefit from it. However, reviewers were undecided on their recommendation for *Voicibel*. Furthermore, the reviewers indicated that they would not pay to use any of these apps.

## Discussion

### Principal Results

We successfully evaluated 3 speech therapy apps: *Loud and Clear*, *Speak Up For Parkinson’s*, and *Voicibel*, and used the MARS framework to assess their engagement, functionality, aesthetics, and information provided. *Loud and Clear* and *Speak Up For Parkinson’s* demonstrated higher quality compared to *Voicibel*.

Speech therapy is a cornerstone of managing these challenges, and mobile apps are emerging as potential aids. While research on their effectiveness is ongoing, there are some exciting options. *Loud and Clear*, *Speak Up For Parkinson’s*, and *Voicibel* offer targeted exercises to improve volume and overall speech quality.

While identifying apps, we initially sought to include those available on the Apple App Store and Google Play, making them more accessible to consumers. However, as we continued, we were unable to find multiple apps that met our criteria. Additionally, we were unable to look at apps available on Google Play due to our lack of access to an Android device. This presented another limitation for the accessibility of this research. We ultimately decided to focus on comparing those available only on the Apple App Store.

The lower ratings for *Loud and Clear* may be attributed to the technological issues found in the app. Similarly, the low ratings for *Speak Up For Parkinson’s* may also be attributed to the lack of access to features due to app malfunctions. *Voicibel* had the highest rating; however, it may be skewed due to the fact that there are only four ratings. *Voicibel* is newer on the market; thus, more time is needed to access the app properly.

*Loud and Clear* aims to strengthen patients’ voices and restore their quality of life through improved communication. The user is guided through a vocal exercise program to regain speech volume and natural melody, thereby increasing intelligibility and restoring communication. The app loads quickly, does not drain the battery, and offers text-to-speech features for accessibility. However, it does not provide real-time feedback on user performance, and the developer collects data from this app for personalized features.

*Speak Up For Parkinson’s* aims to improve the quality of life for the aging population. This app provides speaking tips. While using it, users record themselves saying words and phrases aloud. Then, they watch the video back, observing their faces and how their lips move. This app does not allow users to change the text size or font to accommodate all users. It does not offer special features for people with disabilities, either. The developer does not collect any data from this app.

*Voicibel* aims to examine the user’s voice range. Each exercise allows for customizable goals. These can include reaching a certain intensity or performing within a range. The app does not allow for any visual customization, such as text size and font. This makes it less accommodating for users and reduces its accessibility.

Regarding all 3 apps, observing progress is very subjective. It is up to the patient/user to keep track of their improvement. All of the apps are targeted toward the patient rather than the caretaker. Two apps also offer aspects of volume therapy. *Loud and Clear*, with its color-sensitive scale, and *Speak Up For Parkinson’s*, with its color-coded bar, provide an additional tool to practice volume control through virtual means.

While all 3 apps offer valuable functionalities, limitations exist. *Loud and Clear* lacks real-time feedback and collects user data, which may compromise privacy. Feedback could include annunciation techniques with visual mouth movements for patients to practice. The user data collected consist of the email associated with the account and device information. *Speak Up For Parkinson’s* excludes users with visual or dexterity impairments and relies solely on subjective progress tracking. This includes only a volume control meter that allows the user to visualize the loudness of their speech. While the app contains speaking tips in another section, similar to *Loud and Clear*, it does not provide real-time feedback. *Voicibel* also excludes users with visual impairments, with no customization options. Unlike the other apps, it does not provide any speaking tips. The subjective nature of all 3 apps renders the process entirely reliant on the user, with no feedback.

All 3 apps significantly lack a measure of pitch. While *Loud and Clear* and *Speak Up For Parkinson’s* provide a volume bar, they lack features related to pitch. *Voicibel* only provides a dichromatic measure to check the range. Volume measures the loudness of sound based on the amplitude of sound waves, whereas pitch measures sound frequency [17]. Providing a feedback mechanism for both measures would significantly enhance the effectiveness of virtual therapy.

Additionally, the apps target only patients, neglecting the role that caregivers can play. Future research may include the role caregivers play in speech therapy. To improve, both apps could incorporate real-time feedback, offer opt-out options for data collection, and integrate accessibility features. Caregiver features, objective progress tracking metrics, and potential social or telehealth functionalities could further enhance their effectiveness in empowering people with PD to improve communication skills and their quality of life. Maas et al [18] performed a randomized controlled trial for speech therapy in PD patients. Their study includes the role of the caregiver when necessary, such as providing exercise cues or acting as a conversation partner in severe cases. Caregiver features for PD-specific apps could incorporate similar aspects. Objective progress tracking metrics could include a rating scale of volume and pitch during the performed sessions compared to previous sessions. This would allow users to track their progress and share it with their health care provider if necessary. This could be incorporated with telehealth functionalities, allowing users to share their progress data with their provider. Baseline measurements could be taken to observe the effectiveness of speech therapy. These could include the user-performing tasks such as maximum repetition rate, maximum phonation rate, maximum phonation volume, and fundamental frequency range [19].

Engagement was the lowest for all 3 apps. This showed that the app allowed for basic customization to function adequately and had basic interactive features that also functioned adequately. In contrast, functionality was the highest for all 3 apps. This was related to their performance, ease of use, navigation, and gestural design.

Additionally, all 3 apps had moderately high aesthetics and information scores. *Loud and Clear’s* rating indicated that the app was visually appealing, had high-quality/resolution graphics, and included specific and measurable goals. *Speak Up For Parkinson’s* ratings also demonstrated visually appealing graphics and included highly relevant, appropriate, coherent, and correct information. *Voicibel’s* median ratings showed that the app provided relevant information and displayed stylistic consistency. The SDs show how broadly distributed the sample is. When looking at mean and median ratings, the results show how close the results are.

Although some apps received high MARS scores, the average subjective quality ratings for engagement, functionality, aesthetics, and information suggest that these objective assessments did not fully translate into a positive user experience. While these apps promote awareness for PD and provide a different avenue for speech therapy, they are unable to showcase high user satisfaction. This can be attributed to the subjective nature of a person’s perception of these apps.

IRR agreement scores allowed us to measure the different interpretations of the apps by various reviewers within the MARS framework. This process allowed us to ensure reliability and validity while using a subjective evaluation approach [20]. *Voicibel* had the highest IRR agreement at 0.83 for the engagement section despite receiving the lowest median rating. A high agreement score indicated that the agreement result was not significantly influenced by individual rater bias. Variability between the low agreement for the aesthetics section and the high median rating can be attributed to the subjective nature of this criterion. This reflects that raters may share similar perceptions but differ in the magnitude of scores. Furthermore, our calculation of agreement scores was restrictive, since a difference of less than 0.5 in MARS scores was considered agreement. This narrow agreement band may contribute to some of the disagreement between raters and is correlated with a higher SD of the values. It is important to notice that differences in median and mean are within the agreement range.

Some sources of disagreement between raters may be attributed to the lack of clear definitions for each grade, leading to subjective grading and disagreement. Additionally, due to the small number of raters, the scale became less reliable. With one disagreement, the reliability becomes skewed. The increase in agreement between raters amplifies the reliability of the assessment. With fewer raters, there was a greater impact on the distribution of numbers.

Some sources of disagreement between raters may be attributed to the lack of clear definitions for each grade, leading to subjective grading and disagreement. Additionally, due to the small number of raters, the scale becomes more sensitive to disagreements; with just one disagreement, the reliability scale becomes skewed. Furthermore, some of the variability can be explained by cultural differences and a lack of common ground for interpretation.

A new study evaluated whether speech therapy delivered through mobile apps offers a practical option for patients. Their findings showed 90% adherence among participants who completed the app, ensuring therapeutic effect [21]. This study supports the benefits and feasibility of an at-home speech therapy app.

Speech therapy apps offer promising support for managing speech difficulties in people with PD; however, accessibility limitations can hinder their effectiveness. Patients with PD reported issues such as decreased volume, loudness, weak vocal strength, and imprecise articulation [22]. A significant issue is the need for more customization. This includes text and font control to allow for easier visualization and usability of the apps. PD impacts people differently, and a generic approach might not be practical. Each patient’s severity of the disease and treatment goal may differ and should be considered [18]. Further research can look into app development that incorporates Lee Silverman Voice Therapy techniques. These techniques improve vocal loudness by working on articulation, quality, and intonation [23]. Apps should allow therapists or users to tailor exercises and difficulty to individual needs.

Additionally, most apps currently lack therapist interaction, an essential aspect of speech therapy. Therapist feedback is crucial for making adjustments, maintaining motivation, and ensuring that exercises are performed correctly. They play a vital role in advancing speech production by identifying and addressing communication issues [24]. Cost and device compatibility pose another challenge. Speech therapy apps can be unaffordable, and some require specific devices (such as tablets or smartphones) that users may not be able to afford. For example, in our study, *Speak Up For Parkinson’s* was only compatible with an iPad, Mac, or Apple Vision and not with an iPhone. This creates a barrier for those with limited financial resources or those who are less comfortable with technology. Finally, accessibility features are often lacking. *Loud and Clear*, *Speak Up For Parkinson’s*, and *Voicibel* may need to be optimized for users with motor skill limitations or visual impairments, which are common challenges in PD. Features such as larger text sizes, voice control options, or alternative input methods would significantly improve accessibility. Addressing these accessibility concerns is crucial for speech therapy apps to become more inclusive and reach a broader range of people with PD.

To our knowledge, there is no study in this domain on PD. Compared to previous literature, we found a common result. A study in France evaluated mental health apps on the French App Store using the MARS criteria. Similar to our research, their study also found that functionality was the strength of all of the apps tested, except one [21]. However, they found information to be a weakness, whereas engagement had the lowest rating for our apps. Another study looked at apps for behavioral changes in tobacco cessation. They found the functionality and engagement domains to have higher mean scores than aesthetics and information [22]. A study looking at apps for management of tinnitus also reported the functionality subscale to have the highest median score [23]. These differences may be attributed to the subjective nature of the MARS framework. Furthermore, observing higher functionality in other studies demonstrates that in published apps, this subscale is an expectation. However, other MARS subscales are more subjective due to their behavioral interactions with the user.

### Limitations

Our study only examined free apps available on the Apple App Store. These discounted mobile apps, which may be targeted toward PD, are also available on Google Play. Thus, our study limited our audience to Apple users only. These exclusions may limit the generalizability of the study. Additionally, *Loud and Clear* is only accessible on an iPad. This may also limit accessibility. Since this study only examined free apps, future work could include apps with a subscription fee. For example, *DAF Pro: Slower, clearer speech* is a professional speech therapy app that uses the Delayed Auditory Feedback technique to help with speech. However, this app does require purchasing. Including apps available on the Google Play Store and those requiring a subscription fee would provide a more comprehensive assessment of speech therapy apps available on the market. Additionally, there may be new apps on the market that are not included in our study.

Several steps can be taken to address subjectivity and improve consistency in future evaluations. First, the rating scales could be refined to capture subjective aspects such as engagement and aesthetics. This might involve offering more granular options (eg, “slightly interesting” to “very interesting”) or more explicit definitions of what each score on the scale represents (eg, explaining what level of customization is considered “basic”). Second, facilitating discussions among reviewers about their reasoning behind scores could be helpful. This would allow reviewers to understand each other’s perspectives and refine the framework for future use. Finally, emphasizing objective criteria would benefit information-based questions, such as goal clarity. This might involve breaking down the question into separate ratings for clarity and achievability, focusing on how well-defined the goals are vs how likely they are to be achieved. By acknowledging subjectivity and refining the framework, future MARS evaluations can aim for more consistent and reliable reviewer ratings.

The MARS tool also has limitations. It does not consider app features that contribute to accessibility, such as font size, voice control options, and alternative input methods. These limitations require further study of the tool’s modifications and implications for health apps. Lastly, none of our raters have been diagnosed with PD, so our study is limited in its understanding of the usability of these apps for people living with PD.

### Conclusions

Mobile apps, in general, have created an alternative platform for therapy interventions. We screened existing speech therapy apps using the MARS framework and identified 3 apps for evaluation: *Loud and Clear*, *Speak Up For Parkinson’s*, and *Voicibel*. Through this study, we hope to inform the development and selection of speech therapy apps tailored to the needs of individuals living with PD. Future research with speech therapy apps should evaluate clinical efficacy through randomized controlled trials. This would provide a better measurement of the effectiveness of speech therapy apps on patient outcomes. This would allow greater insight into the evolving technologies and their roles in health care.

## Supplementary material

10.2196/72214 Multimedia Appendix 1 Reviewer Mobile App Rating Scale rating for *Loud and Clear*, *Speak Up For Parkinson’s*, and *Voicibel*.

10.2196/72214 Checklist 1 PRISMA checklist.

## Abbreviations

IRR: interrater reliability
MARS: Mobile App Rating Scale
PD: Parkinson disease
PRISMA: Preferred Reporting Items for Systematic Reviews and Meta-Analyses
