# Supplementary material for: Quality Evaluation Using the Mobile App Rating Scale for Speech Therapy in Parkinson Disease: Systematic Search and Evaluation
Source: JMIR Form Res. 2026 Aug 24;10:e72214. doi: 10.2196/72214 (PMC13502931; doi:10.2196/72214)
Supplement: Supplementary file 1 [file formative-v10-e72214-s001.docx]

Table A1: Reviewer MARS rating for *Loud and Clear*, *Speak Up For Parkinson’s*, and *Voicibel*.

| **Engagement** | | | | |
| --- | --- | --- | --- | --- |
|  | **Reviewer 1** | **Reviewer 2** | **Reviewer 3** | **Reviewer 4** |
| **Loud and Clear** | 3.0 | 3.0 | 4.2 | 3.0 |
| **Speak Up For Parkinson’s** | 2.6 | 3.6 | 4.0 | 4.0 |
| **Voicibel** | 2.8 | 2.8 | 2.6 | 3.2 |
| **Functionality** | | | | |
| **Loud and Clear** | 4.3 | 4.8 | 5.0 | 5.0 |
| **Speak Up For Parkinson’s** | 4.3 | 4.8 | 4.0 | 5.0 |
| **Voicibel** | 5.0 | 4.8 | 3.3 | 5.0 |
| **Aesthetics** | | | | |
| **Loud and Clear** | 3.7 | 4.0 | 5.0 | 4.3 |
| **Speak Up For Parkinson’s** | 2.7 | 4.3 | 3.7 | 4.3 |
| **Voicibel** | 3.7 | 3.0 | 3.3 | 4.7 |
| **Information** | | | | |
| **Loud and Clear** | 4.3 | 4.0 | 4.6 | 4.0 |
| **Speak Up For Parkinson’s** | 4.0 | 3.7 | 4.9 | 4.6 |
| **Voicibel** | 3.9 | 3.3 | 4.3 | 4.3 |
