# Supplementary material for: Quality Evaluation Using the Mobile App Rating Scale for Speech Therapy in Parkinson Disease: Systematic Search and Evaluation
Source: JMIR Form Res. 2026 Aug 24;10:e72214. doi: 10.2196/72214 (PMC13502931; doi:10.2196/72214)
Supplement: Supplementary file 2 [file formative-v10-e72214-s002.docx]

| **Section and Topic** | **Item #** | **Checklist item** | **Location where item is reported** |
| --- | --- | --- | --- |
| **TITLE** | | |  |
| Title | 1 | Identify the report as a systematic review. | Not a formal systematic review; this is an apps review that follows the PRISMA-aligned evaluation in Methods. |
| **ABSTRACT** | | |  |
| Abstract | 2 | See the PRISMA 2020 for Abstracts checklist. | Structured abstract provided. |
| **INTRODUCTION** | | |  |
| Rationale | 3 | Describe the rationale for the review in the context of existing knowledge. | Introduction: Para 1–5, rationale linked to existing therapies and benefits of technology inclusion. |
| Objectives | 4 | Provide an explicit statement of the objective(s) or question(s) the review addresses. | Introduction: Para 6-7 paragraph outlines objectives clearly. |
| **METHODS** | | |  |
| Eligibility criteria | 5 | Specify the inclusion and exclusion criteria for the review and how studies were grouped for the syntheses. | Methods: Inclusion/exclusion criteria detailed for app selection. |
| Information sources | 6 | Specify all databases, registers, websites, organisations, reference lists and other sources searched or consulted to identify studies. Specify the date when each source was last searched or consulted. | Methods: Source includes Apple App Store; January 2024 and July 2025. |
| Search strategy | 7 | Present the full search strategies for all databases, registers and websites, including any filters and limits used. | Methods: App search section provides full list of search terms used. |
| Selection process | 8 | Specify the methods used to decide whether a study met the inclusion criteria of the review, including how many reviewers screened each record and each report retrieved, whether they worked independently, and if applicable, details of automation tools used in the process. | Methods: Two reviewers with healthcare background worked together to screen apps. |
| Data collection process | 9 | Specify the methods used to collect data from reports, including how many reviewers collected data from each report, whether they worked independently, any processes for obtaining or confirming data from study investigators, and if applicable, details of automation tools used in the process. | Methods: Data collected independently by four reviewers; MARS evaluations. |
| Data items | 10a | List and define all outcomes for which data were sought. Specify whether all results that were compatible with each outcome domain in each study were sought (e.g. for all measures, time points, analyses), and if not, the methods used to decide which results to collect. | Not applicable. Methods: did not evaluate outcomes, evaluated MARS, included score dimensions and user ratings. |
|  | 10b | List and define all other variables for which data were sought (e.g. participant and intervention characteristics, funding sources). Describe any assumptions made about any missing or unclear information. | Methods: Data items include app store metadata; assumptions noted for dynamic rating data. |
| Study risk of bias assessment | 11 | Specify the methods used to assess risk of bias in the included studies, including details of the tool(s) used, how many reviewers assessed each study and whether they worked independently, and if applicable, details of automation tools used in the process. | Not applicable; risk of bias assessment not performed for app-based observational data. |
| Effect measures | 12 | Specify for each outcome the effect measure(s) (e.g. risk ratio, mean difference) used in the synthesis or presentation of results. | Not applicable; effect measures not used. |
| Synthesis methods | 13a | Describe the processes used to decide which studies were eligible for each synthesis (e.g. tabulating the study intervention characteristics and comparing against the planned groups for each synthesis (item #5)). | Methods: Apps grouped based on inclusion/exclusion; agreement ratings used to analyze MARS synthesis. |
|  | 13b | Describe any methods required to prepare the data for presentation or synthesis, such as handling of missing summary statistics, or data conversions. | Not applicable; no imputation or conversion required. |
|  | 13c | Describe any methods used to tabulate or visually display results of individual studies and syntheses. | Results: Radar chart used for visualization of app dimensions and multivariate data. |
|  | 13d | Describe any methods used to synthesize results and provide a rationale for the choice(s). If meta-analysis was performed, describe the model(s), method(s) to identify the presence and extent of statistical heterogeneity, and software package(s) used. | Methods: Interrater reliability to look for agreement. |
|  | 13e | Describe any methods used to explore possible causes of heterogeneity among study results (e.g. subgroup analysis, meta-regression). | Not applicable. |
|  | 13f | Describe any sensitivity analyses conducted to assess robustness of the synthesized results. | Not applicable; no sensitivity analyses performed. |
| Reporting bias assessment | 14 | Describe any methods used to assess risk of bias due to missing results in a synthesis (arising from reporting biases). | Not applicable. |
| Certainty assessment | 15 | Describe any methods used to assess certainty (or confidence) in the body of evidence for an outcome. | Interrater reliability to describe any certainty of confidence of the evaluation. |
| **RESULTS** | | |  |
| Study selection | 16a | Describe the results of the search and selection process, from the number of records identified in the search to the number of studies included in the review, ideally using a flow diagram. | Results: PRISMA-style flow diagram provided (Figure 1). |
|  | 16b | Cite studies that might appear to meet the inclusion criteria, but which were excluded, and explain why they were excluded. | Results: Apps excluded based on requiring subscription. |
| Study characteristics | 17 | Cite each included study and present its characteristics. | Results: App characteristics and scores summarized in Results and supplementary table. |
| Risk of bias in studies | 18 | Present assessments of risk of bias for each included study. | Not applicable; no formal bias assessment tool used. |
| Results of individual studies | 19 | For all outcomes, present, for each study: (a) summary statistics for each group (where appropriate) and (b) an effect estimate and its precision (e.g. confidence/credible interval), ideally using structured tables or plots. | Results: Table 2 displays summary statistics. |
| Results of syntheses | 20a | For each synthesis, briefly summarise the characteristics and risk of bias among contributing studies. | Results: Multivariate data visualized in radar charts; bias not assessed. |
|  | 20b | Present results of all statistical syntheses conducted. If meta-analysis was done, present for each the summary estimate and its precision (e.g. confidence/credible interval) and measures of statistical heterogeneity. If comparing groups, describe the direction of the effect. | Results: Median and interrater reliability reported. |
|  | 20c | Present results of all investigations of possible causes of heterogeneity among study results. | Not applicable. |
|  | 20d | Present results of all sensitivity analyses conducted to assess the robustness of the synthesized results. | Not applicable. |
| Reporting biases | 21 | Present assessments of risk of bias due to missing results (arising from reporting biases) for each synthesis assessed. | Reporting bias is present in the interrater reliability applied to the subscales. |
| Certainty of evidence | 22 | Present assessments of certainty (or confidence) in the body of evidence for each outcome assessed. | Interrater reliability to describe any certainty of confidence of the evaluation. |
| **DISCUSSION** | | |  |
| Discussion | 23a | Provide a general interpretation of the results in the context of other evidence. | Discussion: Interprets findings in context of Lee et al. (2025), Rohl et al. (2022), and Church (2021). |
|  | 23b | Discuss any limitations of the evidence included in the review. | Discussion: Limitations include accessibility, sample size, dynamic ratings, small number of raters. |
|  | 23c | Discuss any limitations of the review processes used. | Discussion: Notes limitations of inclusion/exclusion criteria (e.g., lack of transparency, variability in results), the limited number of expert reviewers used for MARS assessments, and subjectivity of MARS. |
|  | 23d | Discuss implications of the results for practice, policy, and future research. | Discussion: Recommends randomized controlled trials to measure effectiveness. |
| **OTHER INFORMATION** | | |  |
| Registration and protocol | 24a | Provide registration information for the review, including register name and registration number, or state that the review was not registered. | Review was not registered. |
|  | 24b | Indicate where the review protocol can be accessed, or state that a protocol was not prepared. | No formal protocol was prepared. |
|  | 24c | Describe and explain any amendments to information provided at registration or in the protocol. | Not applicable. |
| Support | 25 | Describe sources of financial or non-financial support for the review, and the role of the funders or sponsors in the review. | Funding Statement: None declared. |
| Competing interests | 26 | Declare any competing interests of review authors. | Conflict of Interest: None declared. |
| Availability of data, code and other materials | 27 | Report which of the following are publicly available and where they can be found: template data collection forms; data extracted from included studies; data used for all analyses; analytic code; any other materials used in the review. | Data Availability section included; data extraction included in the appendix; no public dataset shared. |

*From:*  Page MJ, McKenzie JE, Bossuyt PM, Boutron I, Hoffmann TC, Mulrow CD, et al. The PRISMA 2020 statement: an updated guideline for reporting systematic reviews. BMJ 2021;372:n71. doi: 10.1136/bmj.n71. This work is licensed under CC BY 4.0. To view a copy of this license, visit <https://creativecommons.org/licenses/by/4.0/>
